# A Molecular Phylogeny of Hemiptera Inferred from Mitochondrial Genome Sequences

**DOI:** 10.1371/journal.pone.0048778

**Published:** 2012-11-08

**Authors:** Nan Song, Ai-Ping Liang, Cui-Ping Bu

**Affiliations:** 1 Key Laboratory of Zoological Systematics and Evolution, Institute of Zoology, Chinese Academy of Sciences, Chaoyang District, Beijing, China; 2 Henan Entry-Exit Inspection and Quarantine Bureau, Jinshui District, Zhengzhou, China; 3 Jiangsu Key Laboratory for Eco-agricultural Biotechnology, Huaiyin Normal University, Huaiyin, China; University of Sydney, Australia

## Abstract

Classically, Hemiptera is comprised of two suborders: Homoptera and Heteroptera. Homoptera includes Cicadomorpha, Fulgoromorpha and Sternorrhyncha. However, according to previous molecular phylogenetic studies based on 18S rDNA, Fulgoromorpha has a closer relationship to Heteroptera than to other hemipterans, leaving Homoptera as paraphyletic. Therefore, the position of Fulgoromorpha is important for studying phylogenetic structure of Hemiptera. We inferred the evolutionary affiliations of twenty-five superfamilies of Hemiptera using mitochondrial protein-coding genes and rRNAs. We sequenced three mitogenomes, from *Pyrops candelaria*, *Lycorma delicatula* and *Ricania marginalis*, representing two additional families in Fulgoromorpha. *Pyrops* and *Lycorma* are representatives of an additional major family Fulgoridae in Fulgoromorpha, whereas *Ricania* is a second representative of the highly derived clade Ricaniidae. The organization and size of these mitogenomes are similar to those of the sequenced fulgoroid species. Our consensus phylogeny of Hemiptera largely supported the relationships (((Fulgoromorpha,Sternorrhyncha),Cicadomorpha),Heteroptera), and thus supported the classic phylogeny of Hemiptera. Selection of optimal evolutionary models (exclusion and inclusion of two rRNA genes or of third codon positions of protein-coding genes) demonstrated that rapidly evolving and saturated sites should be removed from the analyses.

## Introduction

Insect mitochondrial genomes (mitogenomes) are small, double-stranded, circular DNA molecules, ranging in size from approximately 14 to 19 kb. The mitogenome encodes thirty-seven genes (13 protein-coding, 22 transfer RNA, and 2 ribosomal RNA genes), and contains a control region (A+T-rich region) that is thought to play a role in the initiation of transcription and replication, and is a source of length variation in the genome [1], [2]. Complete or nearly complete mitogenome sequences have been increasingly used for phylogenetic analyses above the family level in insects [3], [4]. Mitogenome sequences have able to resolve intraordinal relationships within Coleoptera [5], [6], [7], [8], [9], Lepidoptera [10], [11], [12], Hymenoptera [13], [14], [15], [16], Orthoptera [17], [18], Diptera [19], Neuroptera [20], and Isoptera [21]. Additionally, rearrangements of mitochondrial genes and nucleotide compositional biases can provide useful information for understanding the genetic differences between taxa [22].

Hemiptera is one of the largest insect orders, comprising more than 50,000 described species. The extraordinary diversity in terms of morphology and lifestyle adaptions has long attracted the attention of evolutionary biologists and systematists. For example, some hemipteran species display bizarre morphology, some are brilliantly colored, and some produce cuticular waxes (e.g., the strangely protruding head of *Fulgora laternaria* and the white wax of *Geisha distinctissima*) [23]. *Triatoma dimidiata* is the vector of Chagas disease, which is a predominantly chronic disease affecting millions of people [24]. Due to high reproductive potentials, capabilities of dispersal, and transmission of plant viral diseases, some delphacids have caused considerable damage to grain production and are identified as the causes of rice famines in several Asian countries [25].

Hemipterans have been traditionally recognized as comprising three major groups: Heteroptera (true bugs, including Coleorrhyncha), Sternorrhyncha (aphids, scale bugs, whiteflies, and psyllids), and Auchenorrhyncha (planthoppers, leafhoppers, spittlebugs, and cicadas) [26]. Sternorrhyncha and Auchenorrhyncha were once considered to form the order “Homoptera”, with Heteroptera being classified as an independent order [27], [28]. In “Homoptera”, the monophyly of superfamilies is well supported by morphology [29], [30]. However, the monophyly of “Homoptera” as a whole has been debated. Originally, on the basis of the wing morphology, Linneaus distinguished Homoptera from Heteroptera [31]. Some synapomorphies justifying the monophyly of Homoptera have been proposed [32], [33]. Based on the analyses of morphological data, Hamilton [33] suggested that the clade (Fulgoromorpha,(Sternorrhyncha,Cicadomorpha)) was sister to the clade (Heteroptera,Coleorrhyncha). But recent cladistic analyses using both morphological characters and partial *18S rDNA* sequence data indicated that “Homoptera” is not a monophyletic group [27], [31], [34], [35].

The hemipteran infraorder Fulgoromorpha includes more than 9000 described species in ∼20 families, and all taxa in Fulgoromorpha are terraneous and plant-feeding. The monophyly of Fulgoromorpha is well supported by morphological and molecular data [36], [37]. However, the taxonomic position of Fulgoromorpha within Hemiptera is still controversial. Previous morphological studies suggested that Fulgoromorpha and Cicadomorpha formed Auchenorrhyncha, and that Auchenorrhyncha is more closely related to Coleorrhyncha and Sternorrhyncha than to Heteroptera [38]. Cobben [39] suggested that both Heteroptera and Fulgoromorpha form the sister clade to (Sternorrhyncha,Cicadomorpha) according to a cladistic study of morphological traits. Hamilton [33] examined the phylogenetic affiliations using mouthparts and features of the head and proposed (Fulgoromorpha,(Sternorrhyncha,Cicadomorpha). Emel’yanov [40] placed the clade (Cicadelloidea + Fulgoroidea) in a sister position to the clade (Cercopoidea + Cicadoidea) and suggested that Fulgoromorpha is closer to Cicadomorpha than to other hemipterans. Although the position of Fulgoromorpha is crucial in determining the phylogenetic framework for Hemiptera, few mitogenome studies have addressed this. Fragments of the mitochondrial genes encoding 16S rRNA, 12S rRNA, *cytb*, and *cox1* of some planthopper species [37], [41], [42], [43], [44], [45], and mitogenomes from three fulgoroid species [46], [47], [48] have already been sequenced and utilized for phylogenetic studies. However, these studies usually have neither sufficient genetic information nor broad taxonomic samples.

Here, we describe three new complete mitogenome sequences, which add a major group (Fulgoridae) and a second representative of the highly derived clade Ricaniidae. In addition, we analyzed all 49 available complete or nearly complete mitogenomes from Hemiptera, with the aim of estimating a phylogeny of the order.

## Materials and Methods

### Ethics Statement

No specific permits were required for the insect specimens collected for this study in China. These specimens were collected on the roadside. The field studies did not involve endangered or protected species. *Pyrops candelaria*, *Lycorma delicatula*, and *Ricania marginalis* are all common fulgoroid species in China and are not included in the “List of Protected Animals in China”.

### Insects

Adult specimens of *Pyrops*, *Lycorma*, and *Ricania* were collected in Fujian, Henan, and Zhejiang provinces, China, respectively. They were preserved in 100% ethanol and stored at −80°C in the Key Laboratory of Zoological Systematics and Evolution, Institute of Zoology, Chinese Academy of Sciences. Flies were identified by Nan Song with reference to Chou et al. [49].

### DNA Extraction, PCR, Cloning, and Sequencing

After an examination of external morphology for identification, the muscle tissue under the pronotum of each specimen was used for DNA extraction. A modified method of salt-extraction protocol [50] was employed to extract DNA.

The whole mitogenome was amplified in overlapping PCR fragments. Initial PCR primers are based on Simon et al. (2006) [51]. Short regions within individual genes were amplified using QIAGEN Taq DNA polymerase (QIAGEN, China) in PCR reaction under the following conditions: 5 min at 94°C, followed by 30 cycles of 50 s at 94°C, 50 s at 50°C, and 1–3 min at 72°C. The final elongation step was continued for 10 min at 72°C. The sequences obtained from these regions were then used to design specific primers for long PCRs that allowed us to link all of the shorter regions. The large fragments (>2000 bp) were obtained using the QIAGEN Long Taq DNA polymerase (QIAGEN, China) under the following conditions: 2 min at 96°C, followed by 30 cycles of 10 s at 98°C, and 3 min at 68°C. The final elongation was continued for 10 min at 72°C. These PCR products were analyzed by 1.0% agarose gel electrophoresis.

PCR products of ∼1200 bp were directly sequenced after purification. Whereas products of 1.2–3 kb were cloned into pBS-T Easy vector (QIAGEN, China), and the resultant plasmid DNA was isolated using the TIANprp Midi Plasmid Kit Purification System (QIAGEN, China) and sequenced by means of primer walking. DNA sequencing was performed using BigDye terminator chemistry and ABI 3730xl Genetic Analyzer (PE Applied Biosystems, USA). After the entire mitogenome for *Pyrops* had been sequenced, the primers were reused to amplify other species; however, some primers were redesigned for efficient sequencing because of minor sequence differences between species.

### Sequence Assembly, Annotation, and Analysis

Raw sequence files were proofread and aligned into contigs in BioEdit 7.0.5.3 [52]. Contig sequences were checked for ambiguous base calls, and only non-ambiguous regions were used for annotation. Sequence alignment, genome assembly, and calculations of nucleotide composition were all conducted with MEGA 5 [53]. The 22 tRNA genes were identified using the tRNAScan-SE server [54]. The tRNAs not found by tRNAScan-SE were identified through comparison with the regions coding these tRNAs in other insects. Protein-coding genes and rRNA genes were determined by comparison with those of published insect mitochondrial sequences. Potential secondary structure folds in the A+T-rich region of the genome were predicted using Mfold 3.2 [55]. New mitogenome sequences obtained in this study were deposited in GenBank under accession numbers FJ006724, EU909203, and JN242415 for *Pyrops*, *Lycorma*, and *Ricania*, respectively (Table S1).

### Phylogenetic Analyses

We conducted phylogenetic analyses using all of the currently available mitogenomes of Hemiptera along with the three newly sequenced ones (Table S1).Three orthopterans and a psocid served as outgroups. The orthopterans form a basal lineage and the psocid is in a sister clade to Hemiptera.

The nucleotide sequences of 13 protein-coding genes and 2 rRNA genes were used to reconstruct the phylogenetic relationships in Hemiptera. All protein-coding genes were aligned at the amino acid level using the default settings in ClustalW (as implemented in MEGA 5). The alignments were back-translated into the corresponding nucleotide sequences. The stop codons of protein-coding genes were excluded when aligned. Two rRNAs were respectively aligned in MEGA 5 as nucleotides. Ambiguously aligned regions of protein-coding genes and rRNA genes were checked by eye. Potential saturation in each protein-coding gene and rRNA was individually assessed, with transitions and transversions plotted against corrected genetic distance using DAMBE 5.1.2 [56]. Both average p-distances and pairwise relative -rates were calculated by PHYLTEST [57].

To study the effects of different data and different inference methods on the phylogenetic estimate, we constructed five datasets with varying gene content: (1) all protein-coding genes plus two rRNAs (ALL_123); (2) protein-coding genes excluding third codon positions, plus two rRNAs (ALL_12); (3) all protein-coding genes alone (PCG-123); (4) protein-coding genes excluding third codon positions (PCG_12); and (5) two rRNAs alone (rRNAs).

Phylogenetic trees were estimated from each data set using maximum parsimony (MP), maximum likelihood (ML). and Bayesian inference (BI). MP analyses were performed using PAUP* 4.0b10 [58], with gaps treated as missing data. A total of 1,000 random-addition searches using tree-bisection-reconnection were performed for each MP analysis. Bootstrap support was calculated from 1000 bootstrap replicates with 100 random additions per replicate in PAUP*. Tree statistics were also calculated in PAUP* (Table 1). All four outgroup taxa, partial outgroup (psocid only), or long-branch taxa were respectively removed from MP analyses to search for potential long-branch attraction artifacts.

**Table 1:** Tree statistics for parsimony analyses.

Data set	Total no. characters analyzed	No. of variablecharacters	No. of parsimony-informativecharacters	CI^a^	RI^b^
ALL_123	13378	11473(86%)	10489(78%)	0.215	0.345
ALL_12	9643	7746(80%)	6797(70%)	0.244	0.395
PCG_123	11205	9525(85%)	8791(78%)	0.210	0.337
PCG_12	7470	5798(78%)	5099(68%)	0.244	0.396
rRNAs	2173	1948(89%)	1698(78%)	0.249	0.414

a CI: consistency index;

b RI: retention index.

ML analyses were conducted using the program TreeFinder [59]. For each dataset, the GTR+I+G model was identified as the best-fit one under the Akaike information criterion (AIC) by the “model proposer” in TreeFinder. For the datasets with 3rd codon positions, each codon position was treated as a separate partition. A search-depth level of 2 was selected and bootstrap analysis was performed with 1000 replicates.

Bayesian analyses were conducted with MrBayes 3.2 [60]. Prior to the analyses we tested for the appropriate nucleotide substitution model via AIC with MrModeltest v2.3 [61] for each gene, for each codon and for all concatenated datasets (Table S2). GTR+I+G were estimated as the best-fit substitution model for all partitions. Each analysis comprised four independent Markov chains of 3∼20 million generations each, sampling every 100 generations, and the first 25% discarded as burn-in. The datasets ALL_123 and PCG_123 were partitioned first by gene, then by codon position. All model parameters were unlinked across partitions. Markov chain stationarity was considered to be reached when the average standard deviation of split frequencies fell below 0.01 and potential scale reduction factor values approached 1.0 [62].

## Results

### Genome Features

**Figure 1:**
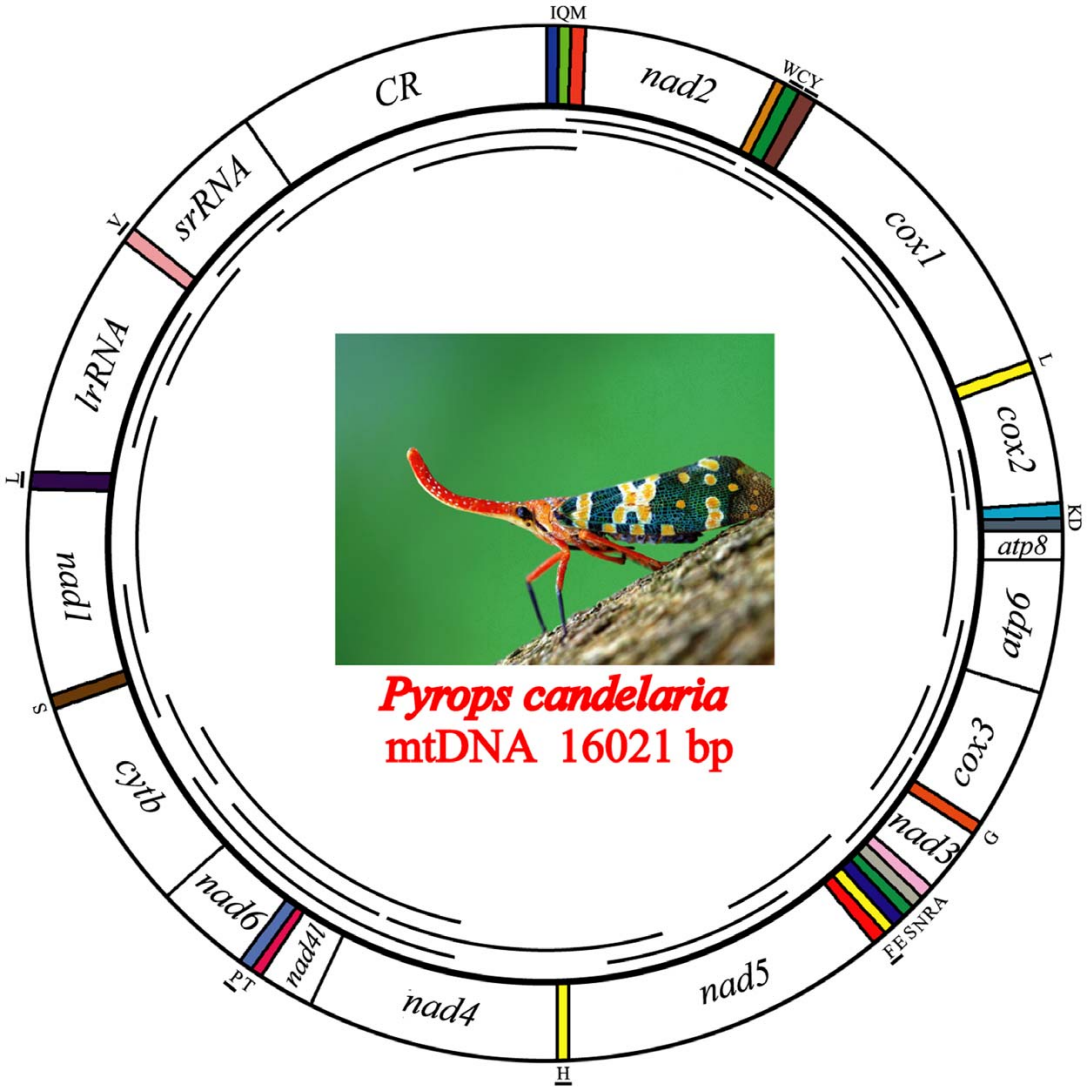
Graphical representation of the mitochondrial genome of *Pyrops candelaria*. Lines within the circle represent the amplification products. The other two fulgoroid species (*Lycorma delicatula* and *Ricania marginalis*) sequenced in this study have the same mitogenome structure as *Pyrops*.

The complete mtDNA sequences of *Pyrops*, *Lycorma*, and *Ricania* were determined to be 16021 bp, 15946 bp, and 15698 bp in size, respectively. The three genome sizes are well within the observed range of insect mitogenomes (14–19 kb) and the length variation occurs in the A+T-rich regions, which range from 1324 to 1642 nucleotides. Like other insect mitogenomes, three fulgoroid mitogenomes contain the typical 13 protein-coding genes (PCGs), 22 tRNAs, two rRNAs, and A+T-rich region (Fig. 1). The position and orientation of the mitochondrial genes are the same as those found in the putative ancestral insect mitogenome [62]. A summary of the mitochondrial genes of *Pyrops*, *Lycorma*, and *Ricania* is given in Table S3.

The nucleotide composition of all three species is significantly biased towards A and T, at 74.3%, 76.4%, and 76.2% respectively, apportioned as follows: protein-coding genes 72.6%, 74.7%, and 75.0%; rRNA genes 75.6%, 77.5%, and 77.6%; and A+T-rich region 83.8%, 83.7%, and 82.3% (Table 2). The nucleotide guanine (G) is the least used, with the frequencies ranging from 8.5–10.3%. With regard to the protein-coding genes, different codon positions have different nucleotide frequencies. The A+T content at the third codon position is higher than that at the first or second codon position. All of the majority-strand genes favor A and C (A-skew and C-skew are 0.24–0.28 and 0.20–0.28, respectively).

**Table 2:** Nucleotide composition for features in the mitogenomes of *Pyrops*, *Lycorma and Ricania.*

genome feature	*Pyrops*					*Lycorma*					*Ricania*				
	%A	%T	%G	%C	Total	%A	%T	%G	%C	Total	%A	%T	%G	%C	Total
All sites	47.7	26.6	10.3	15.3	16021	48.7	27.7	8.5	15.1	15946	47.1	29.1	9.4	14.5	15698
Major-strand protein-coding genes	41.4	29.5	11.5	17.6	6738	42.6	30.8	9.2	17.4	6738	40.9	33.0	10.4	15.7	6741
1st codon position	46.6	23.0	16.3	14.1	2246	45.9	24.7	15.2	14.2	2246	45.1	25.6	15.7	13.6	2247
2nd codon position	22.6	42.3	11.8	23.2	2246	21.7	43.8	11.1	23.4	2246	23.1	43.0	11.1	22.8	2247
3rd codon position	55.1	23.2	6.2	15.5	2246	60.2	23.9	1.4	14.5	2246	54.4	30.4	4.4	10.8	2247
Minor-strand protein-coding genes	18.3	57.0	15.2	9.4	4254	19.1	58.5	14.9	7.4	4212	20.4	56.4	13.8	9.4	4197
1st codon position	20.7	52.0	17.5	9.8	1418	21.2	52.5	17.7	8.5	1404	21.7	50.2	17.9	10.2	1399
2nd codon position	16.9	55.6	16.2	11.3	1418	16.9	55.7	16.7	10.8	1404	17.1	55.8	14.6	12.5	1399
3rd codon position	17.3	63.5	12.0	7.2	1418	19.3	67.4	10.4	2.9	1404	22.4	63.2	9.0	5.4	1399
J-strand tRNA	44.0	30.6	13.3	12.1	909	45.5	31.5	11.6	11.4	907	44.3	33.0	11.7	11.0	909
N-strand tRNA	31.8	41.4	16.4	10.4	531	31.2	43.2	16.5	9.1	516	31.5	41.2	18.3	9.1	515
rRNAs	26.9	48.7	15.2	9.2	1931	27.9	49.6	14.3	8.2	1932	49.3	28.3	8.1	14.3	1952
A+T-rich region	50.8	33.0	7.9	8.4	1592	50.3	33.4	7.0	9.3	1642	48.6	33.7	5.6	12.1	1324

We determined the codon usage of 13 protein-coding genes (Table 3) and found that nine codons (AAT-Asn, TTT-Phe, ATT-Ile, TTA-Leu, AAA-Lys, ATA-Met, TCA-Ser, ACA-Thr, TAT-Tyr) are most frequently used in the three fulgoroids. All of these codons have an A and/or T at the third codon position. This codon bias is also caused by the A+T-rich composition of the mitogenome.

**Table 3:** Codon usage table for *Pyrops*, *Lycorma* and *Ricania* mitochondrial DNA.

a.a.	codon	number			a.a.	codon	number		
		*Pyrops*	*Lycorma*	*Ricania*			*Pyrops*	*Lycorma*	*Ricania*
Ala	GCG	3	1	2	Pro	CCG	8	1	2
	GCA	52	58	60		CCA	45	78	69
	GCU	21	23	31		CCU	42	36	40
	GCC	18	14	5		CCC	34	16	14
Cys	UGU	55	53	43	Gln	CAG	12	7	14
	UGC	9	7	6		CAA	47	50	45
Asn	AAU	97	115	125	Trp	UGG	14	6	10
	AAC	55	35	36		UGA	74	78	69
Asp	GAU	49	44	40	Arg	CGG	5	3	3
	GAC	24	21	18		CGA	24	24	25
Glu	GAG	17	9	6		CGU	11	20	14
	GAA	72	70	80		CGC	6	0	3
Phe	UUU	375	391	408	Ser	AGG	14	13	12
	UUC	79	67	57		AGA	69	76	62
His	CAU	33	33	52		AGU	31	24	23
	CAC	33	32	15		AGC	7	2	1
Gly	GGG	37	12	24	Ser	UCG	6	3	11
	GGA	72	99	77		UCA	131	144	141
	GGU	57	65	77		UCU	97	89	85
	GGC	9	1	5		UCC	20	12	22
Ile	AUU	259	274	317	Thr	ACG	6	3	1
	AUC	73	89	62		ACA	122	121	137
Leu	UUG	71	61	61		ACU	49	52	53
	UUA	171	230	229		ACC	25	23	14
Leu	CUG	13	6	8	Val	GUG	15	8	9
	CUA	75	65	55		GUA	83	74	73
	CUU	57	59	75		GUU	72	86	85
	CUC	7	9	13		GUC	13	5	12
Lys	AAG	29	15	23	Tyr	UAU	111	119	100
	AAA	125	143	169		UAC	37	29	35
Met	AUG	60	30	38	End	UAG	1	1	2
	AUA	316	308	245		UAA	10	10	9

### Transfer RNA and Ribosomal RNA Genes

All 22 tRNA coding genes usually found in mitogenome of metazoans are present in these three fulgoroids. The anticodon nucleotides for the corresponding tRNA genes are identical to those of other available hemipteran mitogenomes [46], [47], [48], [63], [64]. All tRNA genes have the typical clover-leaf structure with one exception: tRNA-ser(AGN), in which the dihydrouridine arm formed a simple loop as in some other metazoan species, including most insects [1], [23], [65], [66]. The tRNAs are all found to be between 60 bp and 75 bp in length.

The arrangements of both 16S rRNA and 12S rRNA in the fulgoroid mitogenomes are conserved. They are located between tRNA-Leu(CUN) and tRNA-Val and between tRNA-Val and the A+T-rich region, respectively (Fig. 1). The lengths of 16S rRNA and 12S rRNA are determined to be 1214–1216 bp and 717–736 bp, respectively. These lengths are similar to those of other sequenced fulgorids (1192–1219 bp for 16S rRNA and 711–747 bp for 12S rRNA in *Sivaloka damnosus*, *Geisha*, and *Laodelphax striatellus*) [46], [47], [48].

### A+T-rich Region

The A+T-rich regions can be roughly divided into five sections, which is similar to other fulgoroid species [46], [47], [48]. In the A+T-rich region, the poly-T stretch may be important to the initiation of mtDNA replication. The stem-loop secondary structure was suggested to be the site of initiation of the secondary strand synthesis in *Drosophila* [67]. Such poly-T and stem-loop structures also exist in the fulgoroids’ A+T-rich region. Repetitive sequences have been commonly found in insect mitogenomes [1], [68], and the length variations are due to the variable number of repeat copies [69], [70], [71]. In the six sequenced fulgoroids, all A+T-rich regions contain tandem repeat units. But there are no any similarities in the size and nucleotide composition of repeat units among them.

### Saturation Test

Because saturation in substitutions can have negative effects on phylogenetic inference [72], the levels of saturation in protein-coding genes and mitochondrial rRNA genes were separately explored (Table S4). Xia's saturation idex (Iss) was estimated and compared to the critical values assuming symmetric (Iss.cSym) and asymmetric (Iss.cAsym) topologies, and a P-value was obtained to assess statistical significance [73], [74]. No saturation was detected in the 1st and 2nd codons of protein-coding genes [P(Iss < Iss.c) <0.05]. For the 3rd codon positions, saturation was detected, with the resulting Iss (0.861) higher than Iss.cSym (0.809). We found similar results in comparisons with Iss.cAsym values. With respect to rRNAs, no saturation was detected in the 16S rRNA alignment, but Iss was greater than both Iss.cSym and Iss.cAsym (NumOUT = 16 or 32) in the 12S rRNA alignment. Therefore, there is some saturation in the third codon positions and rRNAs.

### Sequence Diversity and Relative-rates Tests

We calculated average *p*-distances from PCG_123 between major groups using PHYLTEST (Table S5). The average genetic distances between six whiteflies and other hemipteran groups are higher than those without whiteflies involved. The distances between whiteflies and the psocopteran are close to that between whiteflies and aphids. Furthermore, we carried out pairwise relative-rates tests on PCG_123 using PHYLTEST (Table S6). The family Aleyrodoidea displays higher rates of nucleotide change in the mitochondrial protein-coding genes. The hierarchy of the rate of nucleotide substitution was whiteflies >psyllids > fulgoroids > aphids > cicadas > true bugs. This hierarchy did not change whether Orthoptera or Lepidoptera was used as outgroup.

### Phylogenetic Analyses

In this study, we included 49 taxa of Hemiptera representing four higher groups. After alignment and concatenation, the protein-coding genes totalled 11205 bp and the rRNAs (16S rRNA +12S rRNA) 2173 bp (Table 1).

**Figure 2:**
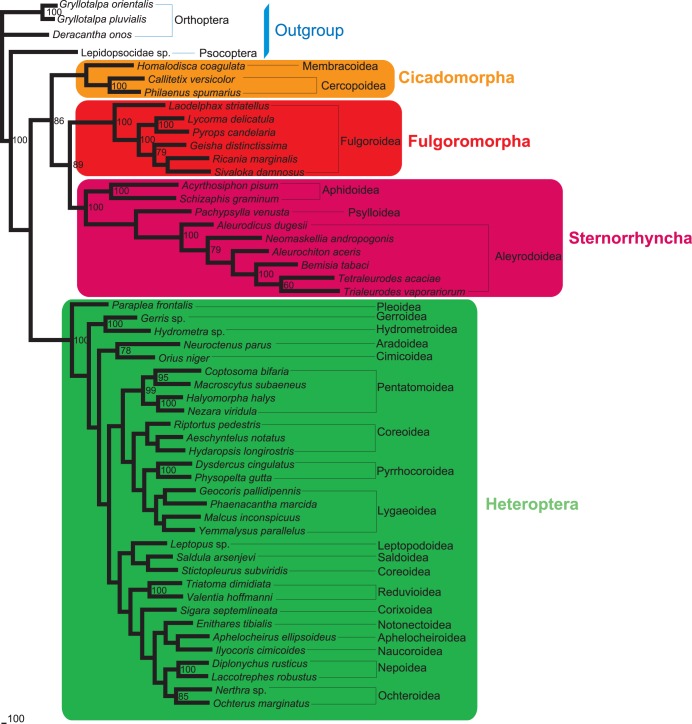
Maximum-parsimony phylogram of 49 hemipterans. Phylogenetic analysis was based on 13 protein-coding genes (only including first and second codon positions) and two rRNA genes. The tree was rooted by Orthoptera and Psocoptera. Only bootstrap support values above 50% are shown.

**Figure 3:**
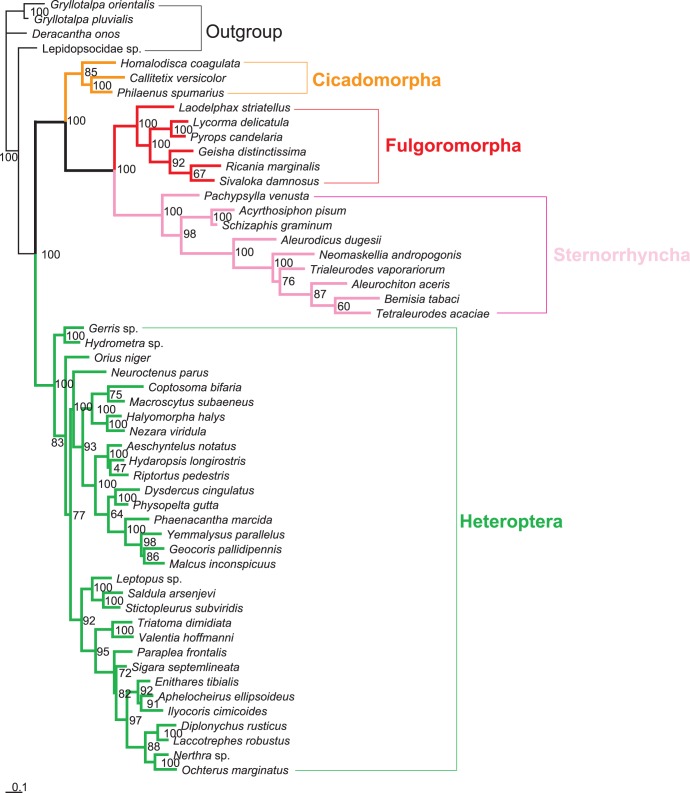
Maximum-likelihood phylogram infered from 13 protein-coding genes (1st and 2nd codon positions) and two rRNA genes. The tree was rooted by Orthoptera and Psocoptera. Only bootstrap support values above 50% are shown.

Five different datasets with three inference methods produced 15 phylogenetic trees. These tree topologies were highly compatible with each other. Fig. 2, 3, 4 illustrates the results of analyses from the ALL_12 dataset.

**Figure 4:**
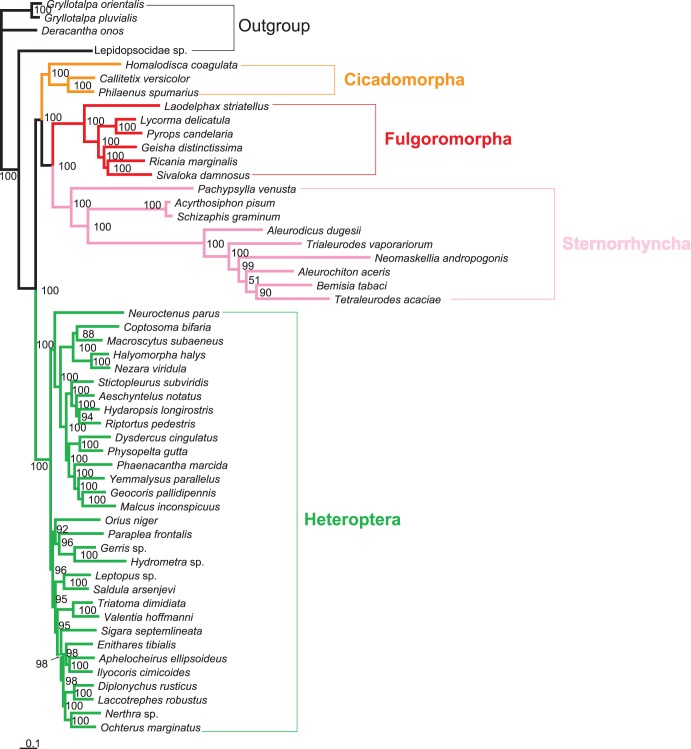
Bayesian phylogram inferred from 13 protein-coding genes (1st and 2nd codon positions) and two rRNA genes. The tree was rooted by Orthoptera and Psocoptera. Only posterior probabilities above 50% are shown.

In most trees provided in this study, Hemiptera was divided into two groups (Cicadomorpha,(Fulgoromorpha,Sternorrhyncha)) and Heteroptera, rendering Auchenorrhyncha paraphyletic. Heteroptera and Sternorrhyncha were well supported (both MP and ML bootstrap support values were 100, and Bayesian posterior probability was 1.00).Within Hemiptera, Fulgoromorpha was closer to Sternorrhyncha than to Heteroptera in 11 trees. In the remaining four trees (MP_ALL_123, MP_PCG_123, MP_rRNAs and BI_rRNAs), the group (Cicadomorpha,Fulgoromorpha) was sister to Heteroptera, and Sternorrhyncha was sister clade to the remaining taxa.

Monophyly at the superfamily level within Sternorrhyncha was well supported in all of the topologies. In the MP trees, Aphidoidea was placed as the sister clade to all other taxa within Sternorrhyncha, whereas Psylloidea was found to be the sister group to the remaining sternorrhynchan lineages. But in all model-based analyses, Psylloidea was recovered as the sister group to all other taxa within Sternorrhyncha, followed by Aphidoidea.

Within the suborder Heteroptera, all the major nodes differed among topologies. Several superfamilies were generally well recovered: Lygaeoidea, Pyrrhocoroidea, Coreoidea, Pentatomoidea, Nepoidea, Ochteroidea, and Reduvioidea.

The interfamilial relationships within Fulgoromorpha were consistently supported. The relationships (Delphacidae,(Fulgoridae,(Flatidae,(Issidae,Ricaniidae))) were resolved in all analyses except for the datasets using rRNAs. The *Laodelphax* was placed as the sister to all other taxa in Fulgoromorpha on all trees, and the monophyly of *Pyrops* + *Lycorma* was also supported irrespective of the analytical method used.

### Effects of Long-branch Attraction

When parsimony and model-based estimates of phylogeny infer different trees, it is desirable to explore the causes of the discrepancy [75]. Long-branch attraction (LBA) is commonly cited as the reason for incongruence between different tree-reconstruction methods [76], [77]. Maximum parsimony is considered susceptible to attraction between long branches [78]. In the present study, six representatives of Aleyrodoidea always display branches significantly longer than other hemipteran species (Fig. 2, 3, 4 and Figure S1). This raised the suspicion that the resulting topologies may be compromised by a long-branch attraction artifact.

**Figure 5:**
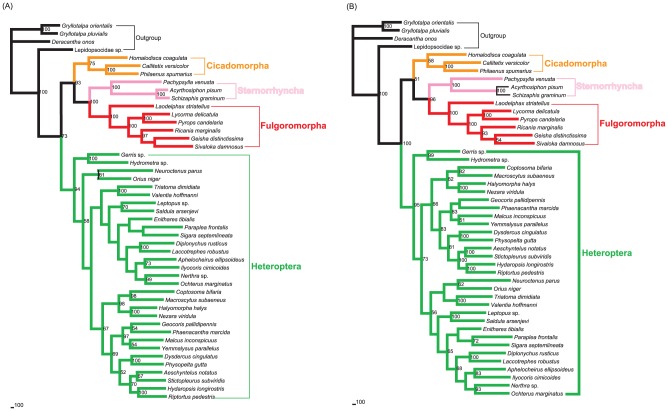
Maximum-parsimony trees from datasets excluding long-branch taxa. (A) Analysis of dataset ALL_123. (B) Analysis of dataset PCG_123. Only bootstrap values above 50% are shown.

**Figure 6:**
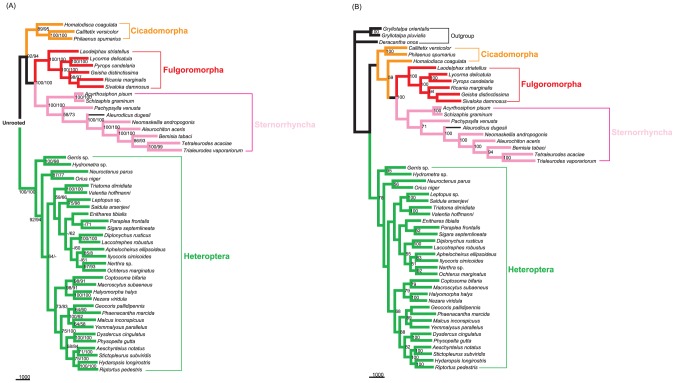
Maximum-parsimony trees from datatsets excluding outgroups. (A) Excluding all four outgroups. Branch support values are given as bootstrap values for the dataset ALL_123 (left) and PCG_123 (right). (B) Outgroups excluding only psocid. Only bootstrap values above 50% are shown.

To test whether LBA affected our phylogenetic analyses, we followed several strategies suggested by Bergsten [79]:

- Datasets without 3rd codon positions. When excluding 3rd codon positions, all analyses provided congruent topologies with high statistical support for major nodes: Fulgoromorpha has a closer relationship to Sternorrhyncha than to other lineages. However, MP analyses including 3rd codon positions produced conflict topologies, with a sister relationship between Fulgoromorpha and Heteroptera. The model-based analyses (ML and BI) yielded the same results as those without 3rd codons.
- Exclusion of long-branch taxa. In MP analyses based on datasets ALL_123 and PCG_123, excluding six taxa with long branches yielded almost identical internal structure of Hemiptera: Fulgoromorpha formed a sister group to Sternorrhyncha with high support values (bootstrap value = 100), and Heteroptera alone constituted a major clade within Hemiptera. The topology was very similar to that obtained with the datasets excluding 3rd codon positions, but most higher taxa were recovered with higher support (Fig. 5).
- Removal of outgroups. MP analyses of datasets ALL_123 and PCG_123, excluding four outgroup taxa, produced an identical tree topology for Hemiptera (Fig. 6). The only difference was that the MP tree on PCG_123 had an increase in support for a few nodes. In our analyses of rRNAs, we found that the outgroup psocid was always nested within the ingroup. Accordingly, we also estimated trees using PCG_123 with the psocid removed. We estimated a congruent ingroup topology with those removing all outgroups.

The analyses performed above indicated that there is a strong attraction of the clade Sternorrhyncha to the outgroups, and this probably led to LBA artifacts.

### rRNA Dataset

The rRNA dataset produced less-resolved topologies. The outgroup Lepidopsocidae sp. tended to group within the ingroup close to Sternorrhyncha in ML and BI analyses. In terms of the family-level relationships in Fulgoromorpha, Flatidae+Ricaniidae formed a moderately supported clade; the position of Issidae varied across different analytical methods. Coreoidea was paraphyletic in the three rRNA analyses and several other datasets that included rRNA (MP_ALL_12, ML_ALL_12, and ML_ALL_123). It is possible that the rRNA data support paraphyly of Coreoidea. Similarly, the monophyly of Cicadomorpha could not be confirmed by the rRNA dataset in MP and ML analyses. These problematic topologies might result from alignment errors or from long-branch attraction.

## Discussion

### Mitochondrial Genome Structure

The start codon for *cox1* is highly variable across insects, and frequently uses noncanonical start codons that code for amino acids other than methionine [80], [81], [82]. However, fulgoroid species do not share this feature. *Pyrops* uses ATC and *Ricania* uses ATA as start codon for *cox1*, whereas conventional methionine (start codon with ATG) is used in *Lycorma*. Although ATG, ATT, ATA, or ATC is universally used as a start codon in mitochondrial protein-coding genes in vertebrates and insects [83], [84], there are apparent exceptions to the ATN rule [1]. For example, GTG has been found as the start codon in some insect mitogenomes: for *atp8* in *Bactrocera dorsalis* [66] and for *nad5* in *Triatoma* [24] and *Pteronarcys princes* [84]. Similarly in the mitogenome of *Pyrops*, we found GTG as start codon for *nad1*.

In accordance with other fulgoroid species, overlapping protein-coding genes are present in *Pyrops*, *Lycorma* and *Ricania*; a 7-bp overlap exists not only between *atp8* and *atp6* but also between *nad4l* and *nad4*. In this case, hairpin structures forming at the 3′ end of the upstream protein’s mRNA may act as a signal for the cleavage of the polycistronic primary transcript [62].

### Phylogenetic Relationships within Hemiptera

Maximum-parsimony analysis, in which all characters were included, produced poorly resolved and poorly supported trees. Excluding third codon positions tended to increase both resolution and support for nodes. This may be attributed to the generally high rate of evolution at third codon positions for Hemiptera. Maximum-likelihood and Baysian analyses produced broadly similar topologies to the MP analyses without 3rd codon positions. Concordant results between the different analytical approaches provide some confidence in the subordinal structure obtained for Hemiptera: (((Fulgoromorpha,Sternorrhyncha),Cicadomorpha),Heteroptera).

The monophyly of Hemiptera is well supported by nucleotide sequences of protein-coding genes, which shows that mitogenome data are very effective in resolving relationships within this group. The relationships (Cicadomorpha,(Fulgoromorpha,Sternorrhyncha)) were consistently recovered by the analyses using datasets ALL_12 and PCG_12. This suggested a paraphyletic Auchenorrhyncha, which has been proposed by previous studies [33], [85], [86].

The monophyletic Fulgoromorpha was well recovered in the analyses of the full dataset (PCGs + rRNAs). The phylogenetic hypothesis of (*Laodelphax*,((*Pyrops,Lycorma*),(*Geisha*,(*Sivaloka*,*Ricania*))) was strongly supported in the analyses without rRNAs. This result was consistent with those of other molecular studies [23]. The rRNA dataset produced slightly differing topologies (Figure S1). Common to the trees was the placement of *Laodelphax* as sister taxon to other fulgoroids, which was in line with previous research [25], [37], [41], [87], [88]. On the other hand, a sister relationship between *Pyrops* and *Lycorma* was consistently resolved in all analyses. This was in accordance with the findings of Yeh et al. [37].

Both the full dataset (ALL_123) and the protein-coding genes (PCG_123) supported a monophyletic Sternorrhyncha. This is in agreement with morphology-based studies [30], [38], [89]. Heteroptera was resolved into two major clades. Among pentatomomorphans, the inferred relationships (Pentatomoidea,(Coreoidea,(Lygaeoidea,Pyrrhocoroidea))) were incongruent with a recent molecular study by Hua et al. [63].

### Long-branch Attraction Artifact

Long-branch attraction refers to the erroneous grouping of two or more unrelated branches as sister groups due to undetected parallel evolution (homoplasy) [90]. These homoplasies are more likely to occur along long branches. Parsimony analysis has been demonstrated to be vulnerable to the high level of homoplasy, and to be particularly sensitive to LBA [79]. In contrast, by correcting for saturation of substitutions, model-based methods may be relatively insensitive to LBA and infer topologies more accurately [79].

In this work, the conflict between parsimony and model-based methods was detected. We followed a similar protocol to that of Bergsten [79] to investigate possible LBA artifacts. Our results showed that the topological affinities (Sternorrhyncha,(Heteroptera,(Cicadomorpha,Fulgoromorpha))) is most likely to be the result of the attraction between the whiteflies and the distant psocid outgroup. This is supported by the fact that these two groups share similar rates of evolution and have less sequence divergence. By contrast, the topologies from MP analyses on datasets without 3rd codon positions (removal of fast evolving sites) and those from the model-based methods (which are less commonly affected by LBA) are correct.

### Mitochondrial rRNA Genes

In addition to the datasets comprising protein-coding genes, we attempted to gain a better resolution of hemipteran relationships using different genes evolving at different rates, 16S rRNA and 12S rRNA. However, these data yielded the worst topologies and the weakest nodal support, indicating that they might be unsuitable for reconstructing the evolutionary relationships of higher taxa in Hemiptera. This may be due to the substitutional saturation observed in mitochondrial rRNA genes. In addition, it was previously seen that mitochondrial rRNA genes displayed significant differences among lineages in their evolutionary rates [90]. Thus, the phylogenetic inferences based on mitochondrial rRNAs were likely to be subject to a LBA artifact, and the lineage leading to Sternorrhyncha might have been incorrectly pulled towards the root of the tree.

### Comparison to Previous Phylogenetic Results

Complete mtDNA sequences can be informative at deep phylogenetic levels [91], and their phylogenetic utility has been demonstrated in several insect orders (see Introduction). Our results show the paraphyly of Auchenorrhyncha, which is consistent with analyses of full-length or partial sequences of nuclear 18S rDNA [87], [92], [93]. The discordance between the trees we present here and those reported in previous molecular phylogenetic studies [27], [92], [93] relates to the position of Fulgoromorpha in Hemiptera. This may be attributed to different methods, molecular markers, or outgroups used among the phylogenetic analyses. In addition, previous molecular studies were mainly based on single 18S rDNA fragments (<1000 bp, which seems to contain insufficient information) and parsimony analysis. Both the short sequences and methods sensitive to LBA may cause problems in resolving phylogenetic relationships at the intraordinal level of insects.

Although it has been used extensively for studies inferring phylogenies, mtDNA has shortcomings that can limit its potential in recovering the phylogenetic signal [94]. In contrast, nuclear genes evolve more slowly, making them effective sources of information for the analysis of deep phylogenetic relationships. Therefore, a combination of mitochondrial genomes and nuclear genes is expected to provide more precise estimates of phylogenetic trees.

### Conclusions

Hemiptera is the largest nonholometabolan insect assemblage and still contains many species that are underrepresented by complete mitochondrial sequences. Although our limited taxon sampling only can provide a preliminary phylogenetic picture of Hemiptera, our result confirms that mitogenome data are very effective in resolving deeper relationships within this order, and the inclusion or exclusion of third codon positions has a strong influence on phylogenetic reconstruction. Moreover, our results are compatible with our previous findings based on more limited taxon sampling [47], [48]. Most topologies inferred in the current study appear to be more consistent with the classical hypothesis by Hamilton [33]. This work has added to current knowledge on the hemipteran phylogeny inferred from mitogenomes. Future research that integrates more taxon sampling, more mitogenome sequences, and data from other molecular markers will provide greater insight into the evolution of Hemiptera.

## Supporting Information

Figure S1 **Tree topologies of MP, ML, and BI inferred from different data partitions except ALL-12.**
(RAR)

Table S1 **List of taxa used in the phylogenetic analysis.**
(XLS)

Table S2 **Nucleotide substitution models selected by MrModelTest2.3 for the data partitions, based on the Akaike Information Criterion.**
(XLS)

Table S3 **Summary of the mitochondrial genes of** ***Pyrops*** **,** ***Lycorma*** **, and** ***Ricania*** **.**
(XLS)

Table S4 **Test of substitutional saturation.**
(XLS)

Table S5 **Average p-distances between major groups in this study.**
(XLS)

Table S6 **Relative-rates test as implemented in PHYLTEST.**
(XLS)

## References

[1] Wolstenholme DR (1992) Animal mitochondrial DNA: structure and evolution. Int Rev Cytol 141: 173–216. 1452431 10.1016/s0074-7696(08)62066-5

[2] Inohira K, Hara T, Matsuura ET (1997) Nucleotide sequence divergence in the A+T-rich region of mitochondrial DNA in *Drosophila simulans and Drosophila mauritiana* . Mol Biol Evol 14: 814–822. 9254919 10.1093/oxfordjournals.molbev.a025822

[3] Nardi F, Spinsanti G, Boore JL, Carapelli A, Dallai R, et al (2003) Hexapod origins: monophyletic or paraphyletic? Science 299: 1887–1889. 12649480 10.1126/science.1078607

[4] Cook CE, Yue QY, Akam M (2005) Mitochondrial genomes suggest that hexapods and crustaceans are mutually paraphyletic. Proceedings of the Royal Society of London Series B 272: 1295–1304. 16024395 10.1098/rspb.2004.3042 PMC1564108

[5] Sheffield NC, Song H, Cameron SL, Whiting MF (2009) Nonstationary Evolution and Compositional Heterogeneity in Beetle Mitochondrial Phylogenomics. Syst Biol 58: 381–394. 20525592 10.1093/sysbio/syp037

[6] Song H, Sheffield NC, Cameron SL, Miller KB, Whiting MF (2010) When phylogenetic assumptions are violated: base compositional heterogeneity and among-site rate variation in beetle mitochondrial phylogenomics. Syst Entomol 35: 429–448.

[7] Timmermans MJ, Dodsworth S, Culverwell CL, Bocak L, Ahrens D, et al (2010) Why barcode? High-throughput multiplex sequencing of mitochondrial genomes for molecular systematics. Nucleic Acids Res 38: e197. 20876691 10.1093/nar/gkq807 PMC2995086

[8] Pons J, Ribera I, Bertranpetit J, Balke M (2010) Nucleotide substitution rates for the full set of mitochondrial protein-coding genes in Coleoptera. Mol Phylogenet Evol 56: 796–807. 20152911 10.1016/j.ympev.2010.02.007

[9] Timmermans MJ, Vogler AP (2012) Phylogenetically informative rearrangements in mitochondrial genomes of Coleoptera, and monophyly of aquatic elateriform beetles (Dryopoidea). Mol Phylogenet Evol 63: 299–304. 22245358 10.1016/j.ympev.2011.12.021

[10] Jiang ST, Hong GY, Yu M, Li N, Yang Y, et al (2009) Characterization of the complete mitochondrial genome of the giant silkworm moth, *Eriogyna pyretorum* (Lepidoptera: Saturniidae). Int J Biol Sci 5: 351–365. 19471586 10.7150/ijbs.5.351 PMC2686093

[11] Chai HN, Du YZ, Zhai BP (2012) Characterization of the Complete Mitochondrial Genomes of *Cnaphalocrocis medinalis* and *Chilo suppressalis* (Lepidoptera: Pyralidae). Int J Biol Sci 2012 8: 561–579. 10.7150/ijbs.3540 PMC3334671 22532789

[12] Kim MJ, Kang AR, Jeong HC, Kim KG, Kim I (2011) Reconstructing intraordinal relationships in Lepidoptera using mitochondrial genome data with the description of two newly sequenced lycaenids, *Spindasis takanonis* and *Protantigius superans* (Lepidoptera: Lycaenidae). Mol Phylogenet Evol 6: 436–445. 10.1016/j.ympev.2011.07.013 21816227

[13] Castro LR, Dowton M (2007) Mitochondrial genomes in the Hymenoptera and their utility as phylogenetic markers. Syst Entomol 32: 60–69.

[14] Cameron SL, Dowton M, Castro LR, Ruberu K, Whiting MF, et al (2008) Mitochondrial genome organization and phylogeny of two vespid wasps. Genome 51: 800–808. 18923531 10.1139/G08-066

[15] Dowton M, Cameron SL, Austin AD, Whiting MF (2009) Phylogenetic approaches for the analysis of mitochondrial genome sequence data in the Hymenoptera - a lineage with both rapidly and slowly evolving mitochondrial genomes. Mol Phylogenet Evol 52: 512–519. 19364540 10.1016/j.ympev.2009.04.001

[16] Kaltenpoth M, Showers Corneli P, Dunn DM, Weiss RB, Strohm E, et al (2012) Accelerated Evolution of Mitochondrial but Not Nuclear Genomes of Hymenoptera: New Evidence from Crabronid Wasps. PLoS ONE 7(3): e32826 doi:10.1371/journal.pone.0032826. 22412929 10.1371/journal.pone.0032826 PMC3295772

[17] Fenn JD, Song H, Cameron SL, Whiting MF (2008) A preliminary mitochondrial genome phylogeny of Orthoptera (Insecta) and approaches to maximizing phylogenetic signal found within mitochondrial genome data. Mol Phylogenet Evol 49: 59–68. 18672078 10.1016/j.ympev.2008.07.004

[18] Ma C, Liu CX, Yang PC, Kang L (2009) The complete mitochondrial genomes of two band-winged grasshoppers, *Gastrimargus marmoratus* and *Oedaleus asiaticus* . BMC Genomics 10: 156. 19361334 10.1186/1471-2164-10-156 PMC2674460

[19] Cameron SL, Lambkin CL, Barker SC, Whiting MF (2007) A mitochondrial genome phylogeny of Diptera: whole genome sequence data accurately resolve relationships over broad timescales with high precision. Syst Entomol 32: 40–59.

[20] Cameron SL, Sullivan J, Song H, Miller KB, Whiting MF (2009) A mitochondrial genome phylogeny of the Neuropterida (lacewings, alderflies and snakeflies) and their relationship to the other holometabolous insect orders. Zool Scr 38: 575–590.

[21] Cameron SL, Whiting MF (2007) Mitochondrial genomic comparisons of the subterranean termites from the Genus *Reticulitermes* (Insecta: Isoptera: Rhinotermitidae). Genome 50: 188–202. 17546084 10.1139/g06-148

[22] Boore JL, Macey JR, Medina M (2005) Sequencing and comparing whole mitochondrial genomes of animals. In Molecular Evolution: Producing the Biochemical Data, Part B Volume 395. San Diego: Elsevier Academic Press Inc. 311–348. 10.1016/S0076-6879(05)95019-2 15865975

[23] Urban JM, Cryan JR (2007) Evolution of the Planthoppers (Insecta: Hemiptera: Fulgoroidea). Mol Phylogenet Evol 42: 556–572. 17011797 10.1016/j.ympev.2006.08.009

[24] Dotson EM, Beard CB (2001) Sequence and organization of the mitochondrial genome of the Chagas disease vector, *Triatoma dimidiata* . Insect Mol Biol 10: 205–215. 11437912 10.1046/j.1365-2583.2001.00258.x

[25] Dyck VA, Thomas B (1979) The brown planthopper problem. In IRRI “Brown planthopper: threat to rice production in Asia”. 3–17. IRRI.Los Banos Philippines, 369.

[26] Carver M, Gross GF, Woodward TE (1991) Hemiptera (bugs, leafhoppers, cicadas, aphids, scale insects, etc.). *In* The insects of Australia, a textbook for students and research workers. Edited by CSIR Organization. Melbourne University Press, Victoria, Australia 429–509.

[27] von Dohlen CD, Moran NA (1995) Molecular phylogeny of the Homoptera: a paraphyletic taxon. J Mol Evol 41: 211–223. 7666451 10.1007/BF00170675

[28] Stewart JB, Beckenbach AT (2005) Insect mitochondrial genomics: the complete mitochondrial genome sequence of the meadow spittlebug *Philaenus spumariuss* (Hemiptera: Auchenorrhyncha: Cercopoidae). Genome 48: 46–54. 15729396 10.1139/g04-090

[29] Kramer S (1950) The morphology and phylogeny of auchenorhynchous Homoptera (Insecta). Ill Biol Monog 20: 1–11.

[30] Hennig W (1981) Insect phylogeny. J Wiley and Sons, New York.

[31] Campbell BC, Steffen-Campbell JD, Sorensen JT, Gill RJ (1995) Paraphyly of Homoptera and Auchenorrhyncha inferred from 18S rDNA nucleotide sequences. Syst Entomol 20: 175–194.

[32] Boudreaux HB (1979) Arthropod phylogeny with special reference to insects. J Wiley and Sons, New York.

[33] Hamilton KGA (1981) Morphology and evolution of the rhynchotan head (Insecta: Hemiptera, Homoptera). Can Entomol 113: 953–974.

[34] Wootton RJ, Betts CR (1986) Homology and function in the wings of Heteroptera. Syst Entomol 11: 389–400.

[35] Zrzavy J (1992,) Evolution of antennae and historical ecology of the hemipteran insects (Paraneoptera). Acta Entomol Bohemoslov 89: 77–86.

[36] Asche M (1987) Preliminary thoughts on the phylogeny of Fulgoromorpha (Homoptera Auchenorrhyncha). In: Proceedings of the 6th Auchenorrhyncha Meeting, Turin, Italy, 7–11 September, 47–53.

[37] Yeh WB, Yang CT, Hui CF (2005) A molecular phylogeny of planthoppers (Hemiptera: Fulgoroidea) inferred from mitochondrial 16S rDNA sequences. Zool Stud 44: 519–535.

[38] Evans JW (1963) The phylogeny of the Homoptera. Annu Rev Entomol 8: 77–94.

[39] Cobben RH (1978) Evolutionary trends in Heteroptera. II. Mouthpartstructures and feeding strategies. Mededelingen Landbouwhogeschool, Wageningen, The Netherlands.

[40] Emel’yanov AF (1987) The phylogeny of the Cicadina (Homoptera, Cicadina) based on comparative morphological data. Trudy Vsesoyuznogo Entomologischeskogo Obshchestva 69: 19–109.

[41] Yeh WB, Yang CT, Hui CF (1998) Phylogenetic relationships of the Tropiduchidae-group (Homoptera: Fulgoroidea) of planthoppers inferred through nucleotide sequences. Zool Stud 37: 45–55.

[42] Dijkstra E, Rubio JM, Post RJ (2003) Resolving relationships over a wide taxonomic range in Delphacidae (Homoptera) using the COI gene. Syst Entomol 28: 89–100.

[43] Dijkstra E, Slotman ME, Post RJ (2006) Resolution of phylogenetic relationships of the major subfamilies of the Delphacidae (Homoptera: Fulgoroidea) using the mitochondrial ribosomal DNA. Insect Sci 13: 167–177.

[44] Ceotto P, Kergoat GJ, Rasplus J-Y, Bourgoin T (2008) Molecular phylogenetics of cixiid planthoppers (Hemiptera: Fulgoromorpha): new insights from combined analyses of mitochondrial and nuclear genes. Mol Phylogenet Evol 48: 667–678. 18539050 10.1016/j.ympev.2008.04.026

[45] Urban JM, Bartlett C, Cryan J (2010) Evolution of Delphacidae (Hemiptera: Fulgoroidea): combined-evidence phylogenetics reveals importance of grass host shifts. Syst Entomol 35: 678–691.

[46] Song N, Liang A-P (2009a) The complete mitochondrial genome sequence of *Geisha distinctissima* (Hemiptera: Flatidae) and comparison with other hemipteran insects. Acta Biochim Biophys Sin 41: 206–216. 19280059 10.1093/abbs/gmp003

[47] Song N, Liang A-P (2009b) Complete mitochondrial genome of the small brown planthopper, *Laodelphax striatellus* (Delphacidae: Hemiptera), with a novel gene order. Zool Sci 26: 851–860. 19968473 10.2108/zsj.26.851

[48] Song N, Liang A-P, Ma C (2010) The complete mitochondrial genome sequence of the planthopper, *Sivaloka damnosus* . J Insect Sci 10: 76. 20673194 10.1673/031.010.7601 PMC3383430

[49] Chou L, Lu JS, Huang J, Wang SZ (1985) Economic insect fauna of China Fasc. 36 Homoptera Fulgoroidea 1–152.

[50] Aljanabi SM, Martinez I (1997) Universal and rapid salt-extraction of high quality genomic DNA for PCR-based techniques. Nucleic Acids Res 25: 4692–4693. 9358185 10.1093/nar/25.22.4692 PMC147078

[51] Simon C, Buckley TR, Frati F, Stewart JB, Beckenbach AT (2006) Incorporating molecular evolution into phylogenetic analysis, and a new compilation of conserved polymerase chain reaction primers for animal mitochondrial DNA. Annu Rev Ecol Evol Syst 37: 545–579.

[52] Hall TA (1999) BioEdit: a user-friendly biological sequence alignment editor and analysis program for Windows 95/98/NT. Nucleic Acids Symp Ser 41: 95–98.

[53] Tamura K, Peterson D, Peterson N, Stecher G, Nei M, et al (2011) MEGA5: Molecular Evolutionary Genetics Analysis using Maximum Likelihood, Evolutionary Distance, and Maximum Parsimony Methods. Mol Biol Evol 28: 2731–2739. 21546353 10.1093/molbev/msr121 PMC3203626

[54] Lowe TD, Eddy SR (1997) tRNAscan-SE: a program for improved detection of transfer RNA genes in genomic sequence. Nucleic Acids Res 25: 955–964. 9023104 10.1093/nar/25.5.955 PMC146525

[55] Zuker M (2003) Mfold web server for nucleic acid folding and hybridization prediction. Nucleic Acids Res 31: 3406–3415. 12824337 10.1093/nar/gkg595 PMC169194

[56] Xia X, Xie Z (2001) DAMBE: Data analysis in molecular biology and evolution. J Hered 92: 371–373. 11535656 10.1093/jhered/92.4.371

[57] Kumar S (1996) PHYLTEST: a program for testing phylogenetic hypotheses, Version 2.0.

[58] Swofford DL (2003) PAUP*. Phylogenetic Analysis Using Parsimony (* and other methods). Version 4 (beta 10). Sinauer Associates, Sunderland, Massachusetts.

[59] Jobb G, von Haeseler A, Strimmer K (2004) TREEFINDER: a powerful graphical analysis environment for molecular phylogenetics. BMC Evol Biol 4: 18. 15222900 10.1186/1471-2148-4-18 PMC459214

[60] Ronquist F, Teslenko M, van der Mark P, Ayres D, Darling A, et al (2012) MrBayes 3.2: Effcient Bayesian phylogenetic inference and model choice across a large model space. Syst Biol 61: 539–42. 22357727 10.1093/sysbio/sys029 PMC3329765

[61] Nylander JAA (2004) MrModeltest v2. Program distributed by the author. Evolutionary Biology Centre, Uppsala University, Uppsala.

[62] Clary DO, Wolstenholme DR (1985) The mitochondrial DNA molecule of *Drosophila yakuba*: nucleotide sequence, gene organization, and genetic code. J Mol Evol 22: 252–271. 3001325 10.1007/BF02099755

[63] Hua JM, Li M, Dong PZ, Cui Y, Xie Q, et al (2008) Comparative and phylogenomic studies on the mitochondrial genomes of Pentatomomorpha (Insecta: Hemiptera: Heteroptera). BMC Genomics 9: 610. 19091056 10.1186/1471-2164-9-610 PMC2651891

[64] Hua JM, Li M, Dong PZ, Cui Y, Xie Q, et al (2009) Phylogenetic analysis of the true water bugs (Insecta: Hemiptera: Heteroptera: Nepomorpha): evidence from mitochondrial genomes. BMC Evolut Biol 9: 134. 10.1186/1471-2148-9-134 PMC2711072 19523246

[65] Beard CB, Mills D, Collins FH (1993) The mitochondrial genome of the mosquito *Anopheles gambiae*: DNA sequence, genome organization, and comparisons with mitochondrial sequences of other insects. Insect Mol Biol 2: 103–124. 9087549 10.1111/j.1365-2583.1993.tb00131.x

[66] Yu DJ, Xu L, Nardi F, Li JG, Zhang RJ (2007) The complete nucleotide sequence of the mitochondrial genome of the oriental fruit fly, *Bactrocera dorsalis* (Diptera: Tephritidae). Gene 396: 66–74. 17433576 10.1016/j.gene.2007.02.023

[67] Clary DO, Wolstenholme DR (1987) *Drosophila* mitochondrial DNA: conserved sequences in the A+T rich region and supporting evidence for a secondary structure model of the small ribosomal RNA. J Mol Evol 25: 116–125. 3116271 10.1007/BF02101753

[68] Zhang DX, Hewitt GM (1997) Insect mitochondrial control region: a review of its structure, evolution and usefulness in evolutionary studies. Biochem Syst Ecol 25: 99–120.

[69] Broughton RE, Dowling TE (1994) Length variation in mitochondrial DNA of the minnow *Cyprinella spiloptera* . Genetics 138: 179–190. 8001785 10.1093/genetics/138.1.179 PMC1206129

[70] Fumagalli L, Taberlet P, Favre L, Hausser J (1996) Origin and evolution of homologous repeated sequences in the mitochondrial DNA control region of shrews. Mol Biol Evol 13: 31–46. 8583904 10.1093/oxfordjournals.molbev.a025568

[71] Wilkinson GS, Mayer F, Kerth G, Petri B (1997) Evolution of repeated sequence arrays in the D-loop region of bat mitochondrial DNA. Genetics 146: 1035–1048. 9215906 10.1093/genetics/146.3.1035 PMC1208033

[72] Swofford DL, Olsen GJ, Waddell PJ, Hillis DM (1996) Phylogenetic Inference. In *Molecular systematics*, 2^nd^ edition, chap. 5, 407–514. Sinauer and Associates, Sunderland, Massachusetts.

[73] Xia X, Li C, Yang Q (2003) Routine analysis of molecular data with software DAMBE. Pp. 149–167 in Yang, Q. ed. Fundamental concepts and methodology in molecular palaeontology. Science Publishers, China.

[74] Xia X, Lemey P (2009) Assessing substitution saturation with DAMBE. Pp. 615–630 in Philippe Lemey, Marco Salemi and Anne-Mieke Vandamme, eds. The Phylogenetic Handbook: A Practical Approach to DNA and Protein Phylogeny. 2nd edition. Cambridge University Press.

[75] Hillis DM, Huelsenbeck JP, Swofford DL (1994) Hobgoblin of phylogenetics? Nature 369: 363–364. 8196763 10.1038/369363a0

[76] Hendy MD, Penny D (1989) A framework for the quantitative study of evolutionary trees. Syst Zool 38: 297–309.

[77] Philippe H, Zhou Y, Brinkmann H, Rodrigue N, Delsuc F (2005) Heterotachy and long-branch attraction in phylogenetics. BMC Evol Biol 5: 50. 16209710 10.1186/1471-2148-5-50 PMC1274308

[78] Ho SYW, Jermiin LS (2004) Tracing the decay of the historical signal in biological sequence data. Syst Biol 53: 623–637. 15371250 10.1080/10635150490503035

[79] Bergsten J (2005) A review of long-branch attraction. Cladistics 21: 163–193. 10.1111/j.1096-0031.2005.00059.x 34892859

[80] Bae JS, Kim I, Sohn HD, Jin BR (2004) The mitochondrial genome of the firefly, *Pyrocoelia rufa*: complete DNA sequence, genome organization, and phylogenetic analysis with other insects. Mol Phylogenet Evol 32: 978–985. 15288070 10.1016/j.ympev.2004.03.009

[81] Junqueira ACM, Lessinger AC, Torres TT, Rodrigues da Silva F, Vettore AL, et al (2004) The mitochondrial genome of the blowfly *Chrysomya chloropyga* (Diptera: Calliphoridae). Gene 339: 7–15. 15363841 10.1016/j.gene.2004.06.031

[82] Kim I, Lee EM, Seol KY, Yun EY, Lee YB, et al (2006) The mitochondrial genome of the Korean hairstreak, *Coreana raphaelis* (Lepidoptera: Lycaenidae). Insect Mol Biol 15: 217–225. 16640732 10.1111/j.1365-2583.2006.00630.x

[83] Fearnley IM, Walker JE (1987) Initiation codons in mammalian mitochondria: differences in genetic code in the organelle. Biochemistry 26: 8247–8251. 2964865 10.1021/bi00399a034

[84] Stewart JB, Beckenbach AT (2006) Insect mitochondrial genomics 2: the complete mitochondrial genome sequence of a giant stonefly, *Pteronarcys princes*, asymmetric directional mutation bias, and conserved plecopteran A+T-region elements. Genome 49: 815–824. 16936790 10.1139/g06-037

[85] Ross HH (1965) A textbook of entomology, 3rd ed. Wiley, New York.

[86] Goodchild AJP (1966) Evolution of the alimentary canal in the Hemiptera. Biol Rev 41: 97–140.

[87] Bourgoin T, SteVen-Campbell JD, Campbell BC (1997) Molecular phylogeny of Fulgoromorpha (Insecta, Hemiptera, Archaeorrhyncha). The enigmatic Tettigometridae: evolutionary affiliations and historical biogeography. Cladistics 13: 207–224. 10.1111/j.1096-0031.1997.tb00316.x 34911231

[88] Yeh WB, Yang CT (1999) Fulgoromorpha phylogeny based on 28S rDNA nucleotide sequence. Chin J Entomol 11: 87–111.

[89] Schuh RT (1979) Review of RH Cobben, Evolutionary Trends in Heteroptera. Part II. Mouthpart-structures and feeding strategies. Syst Zool 28: 653–656.

[90] Felsenstein J (1978) Cases in which parsimony or compatibility methods will be positively misleading. Syst Zool 27: 401–410.

[91] Curole JP, Kocher TD (1999) Mitogenomics: digging deeper with complete mitochondrial genomes. Trends Ecol Evol 14: 394–398. 10481201 10.1016/s0169-5347(99)01660-2

[92] Campbell BC, Steffen-Campbell JD, Gill RJ (1994) Evolutionary origin of whiteflies (Hemiptera: Sternorrhyncha: Aleyrodidae) inferred from 18s rDNA sequences. Insect Mol Biol 3: 73–89. 7987524 10.1111/j.1365-2583.1994.tb00154.x

[93] Sorensen JT, Campbell BC, Gill RJ, Steffen-Campbell JD (1995) Non-monophyly of Auchenorrhyncha (‘Homoptera’), based upon 18s rDNA phylogeny: eco-evolutionary and cladistic implications within pre-Heteropterodea Hemiptera (s.1.) and a proposal for new, monophyletic suborders. Pan-Pac. Entomol. 71: 31–60.

[94] Douglas DA, Janke A, Arnason U (2006) A mitogenomic study on the phylogenetic position of snakes. Zool Scr 35: 545–558.

